# Nutraceutical Profile of “Carosello” (*Cucumis melo* L.) Grown in an Out-of-Season Cycle under LEDs

**DOI:** 10.3390/antiox11040777

**Published:** 2022-04-13

**Authors:** Onofrio Davide Palmitessa, Miriana Durante, Annalisa Somma, Giovanni Mita, Massimiliano D’Imperio, Francesco Serio, Pietro Santamaria

**Affiliations:** 1 Department of Agricultural and Environmental Science, University of Bari Aldo Moro, Via Amendola 165/a, 70126 Bari, Italy; onofrio.palmitessa@uniba.it (O.D.P.); annalisa.somma@uniba.it (A.S.); pietro.santamaria@uniba.it (P.S.); 2 Institute of Sciences of Food Production, National Research Council of Italy, 73100 Lecce, Italy; miriana.durante@ispa.cnr.it (M.D.); giovanni.mita@ispa.cnr.it (G.M.); 3 Institute of Sciences of Food Production, National Research Council of Italy, 70126 Bari, Italy; massimiliano.dimperio@ispa.cnr.it

**Keywords:** cucurbitaceae, tocopherols, polyphenols, mineral profile, carotenoids, chlorophylls, sugars

## Abstract

The world population is projected to increase to 9.9 billion by 2050 and, to ensure food security and quality, agriculture must sustainably multiply production, increase the nutritional value of fruit and vegetables, and preserve genetic variability. In this work, an Apulian landrace of *Cucumis melo* L. called “Carosello leccese” was grown in a greenhouse with a soilless technique under light-emitting diodes (LEDs) used as supplementary light system. The obtained results showed that “Carosello leccese” contains up to 71.0 mg·g^−1^ dried weight (DW) of potassium and several bioactive compounds important for human health such as methyl gallate (35.58 µg·g^−1^ DW), α-tocopherol (10.12 µg·g^−1^ DW), and β-carotene (up to 9.29 µg·g^−1^ DW under LEDs). In fact, methyl gallate has antioxidative and antiviral effects in vitro and in vivo, tocopherols are well recognized for their effective inhibition of lipid oxidation in foods and biological systems and carotenoids are known to be very efficient physical and chemical quenchers of singlet oxygen. Finally, it was demonstrated that the LEDs’ supplementary light did not negatively influence the biochemical profile of the peponids, confirming that it can be considered a valid technique to enhance horticultural production without reducing the content of the bioactive compounds of the fruits.

## 1. Introduction

Due to the continuous increase of the world population, agriculture must multiply production in a sustainable way, improve the nutritional value of fruit and vegetables, and preserve genetic variability. Part of the challenges described in “The Sustainable Development Goals-2030 Agenda” is reaching “Goal 2”, titled “End hunger, achieve food security and improved nutrition and promote sustainable agriculture” [1]. During the research project called BiodiverSO, funded by Apulian Region (Southern Italy) administration under the Rural Development Program 2014–2020, several landraces and wild edible plants were saved from risk of genetic erosion [2]. The preservation of agrobiodiversity represents a key point in assuring the adaptability and resilience of agroecosystems to the global challenge that we will face in the near future of producing more and better food in a sustainable way [2]. Landraces may represent a valid alternative to hybrid genotype cultivation due to their characteristics: (i) high nutritional value; (ii) lower water requirement compared to modern varieties; (iii) good adaption to pests and poor soils [3]. One of the most representative examples of Apulian vegetable biodiversity is the species *Cucumis melo* L. (melon) of the *Cucurbitaceae* family [4]. Apulia is an important secondary center of diversity for melon [5] and one of the most appreciated Apulian melons is “Carosello”. This melon belongs to *Cucumis melo* L. subsp. *melo* conv. *flexuous* [6] and it is a herbaceous plant [7] whose fruits are typically consumed at an immature stage instead of cucumbers due to their superior quality profile [8].

Recently, a landrace of ”Carosello” called “Carosello leccese” was grown in the greenhouse with soilless system using a supplementary light technique [4,9]. In these studies, Somma et al. [4] used morphophysiological descriptors to distinguish “Carosello” from “Barattiere” (another landrace of immature melon) and to define the optimal crop operations to obtain the highest yield. Palmitessa et al. [9], for the same landrace, studied the effects of light-emitting diode (LED) technology on the morphology, yield, and total content of bioactive compounds. Nevertheless, up to now, no studies have been conducted to define the antioxidant compounds (polyphenols, tocopherols, and carotenoids) or mineral profiles of landraces of “Carosello” fruits in an out-of-season cycle under LEDs used as supplementary light. However, it is very important to address this issue in order to ascertain if the experimental growing conditions and out-of-season cultivation could affect the profile of these nutritionally important compounds. At the same time, in recent years, scientific research about the effects of LEDs used as artificial light on the growth of plants and fruit/vegetables quality has been very relevant. The oldest artificial light technologies, such as high-pressure sodium (HPS), are mainly concerned with regions located at extreme latitudes [10]. However, the fast development of LED technology allows the growth of vegetables in indoor conditions [11] or the improvement of the quality and the quantity of horticultural production during the fall–winter period in the Mediterranean basin [12,13]. Furthermore, with LED technology, it is possible to customize the light spectra composition to have specific effects on plant morphology [14] and physiology [15] or on the antioxidant content of horticultural product, such as polyphenols [16], carotenoids [17], ascorbic acid [18], and other bioactive compounds [19,20].

Recently, Vella et al. [21], studying the polyphenols profile of muskmelon (*C. melo* L.) fruits, reported seven phenolic compounds: gallic acid, chlorogenic acid, rutin, ferulic acid, ellagic acid, quercetin, and kaempferol. Differently to muskmelon, it is worth considering that, being harvested at immature stage, “Carosello” has immature and inconsistent seeds that can be ingested without discomfort and increase the nutritional value of the fruits [22]. Indeed, seeds of the *Cucurbitaceae* family are rich in protein and minerals, such as copper, phosphorus, zinc, iron, and magnesium. They are also a good source of carotenoids and tocopherols, particularly α-tocopherol [23]. Tocopherols, together with tocotrienols, are part of the vitamin E family. α-tocopherol deficiency was demonstrated to be correlated to ataxia in humans [24].

*Cucurbitaceae* fruits contain α-carotene, β-carotene, lutein, and zeaxanthin, which are very well known for their potential role as antioxidants in human health [25].

So far, no specific study has been conducted on the biochemical profile of “Carosello” fruits, but promising expectations can be derived from our previous study [9] and other studies in which it was stated that these fruits have a high potassium and low reducing sugar and sodium contents [26] and do not contain cucurbitacin (highly oxidized tetracyclic triterpenes), the molecule responsible for the bitter taste of cucumber [27]. Therefore, also considering the promising results that we have obtained with the out-of-season production of tomatoes through the use of LEDs in Mediterranean greenhouses [28] and after demonstrating that LEDs increase both the mineral profile and α-tocopherol content of the tomato fruits [18], the aim of this research was to evaluate the polyphenols, tocopherols, carotenoids, chlorophylls, and minerals profile of “Carosello leccese” fruits to evaluate whether the supplementary light of the LEDs might influence the biochemical composition of the bioactive compounds of the fruits.

## 2. Materials and Methods

### 2.1. Experimental Set-Up

The experiment was performed from September to November 2020 at the experimental farm “La Noria” of the Institute of Sciences of Food Production, National Research Council [Mola di Bari, Italy (41.062156° N, 17.066914° E)] in an unheated polymethacrylate greenhouse with a maximum height of 4.5 m. Plants were cultivated on a surface of 80 m^2^ with a soilless technique and the experimental design was a randomized block. More detailed information about experimental set-up and agronomic technique has been described by Palmitessa et al. [9].

### 2.2. Light-Emitting Diodes (LEDs) Application

**Figure 1:**
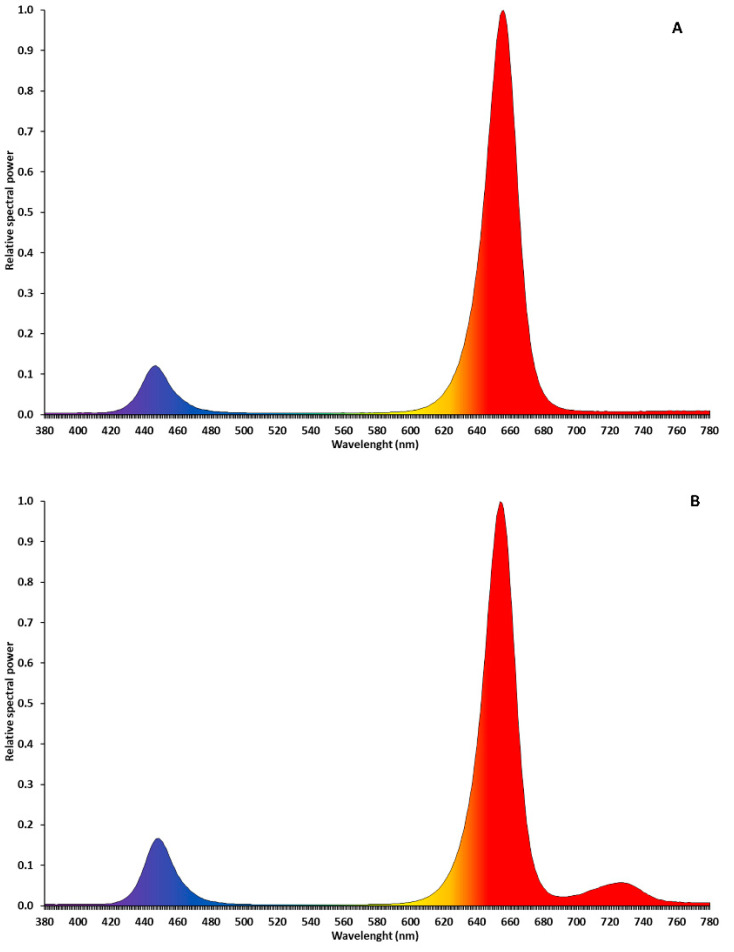
Relative spectral power of LEDs fixtures R + B (**A**) and R + B + FR (**B**). Light spectra were measured with spectrophotometer LI-180 (LI-COR, Lincoln, NE, USA).

LED interlight fixtures (“GreenHouse Interlight” produced by C-Led, Imola (BO), Italy) were used as a supplementary light (SL) system to supply 170 µmol·m^−2^·s^−1^ of extra photosynthetic photon flux density (PPFD). Two light spectra were tested: red (660 nm) + blue (440 nm) (R + B) and red + blue + far red (730 nm) (R + B + FR; Figure 1) Solar light (NL, no supplementary lighting, three benches) was used as control. The interlight modules were placed 25 cm below the apical meristem and the height of the LEDs was adjusted once per week to maintain a constant distance between the apical meristem and the fixtures to ensure uniform light distribution and intensity. Two modules for each experimental unit were used, covering a linear surface of almost five meters. The light treatments started at the transplant date with the LED modules above the apical meristems (the plants were 15 cm tall) and continued until the end of the crop cycle. The SL was operated automatically by a system composed of a CR1000 datalogger (Campbell Scientific, Logan, UT, USA) and a quantum sensor (LI-190R, LI-COR, Lincoln, NE, USA). For 16 h·d^−1^ (from 5:00 to 21:00), the system measured incoming sunlight in the greenhouse at four-second intervals and turned on the LED light bars whenever the ambient PPFD dropped below 200 µmol·m^−2^·s^−1^. More detailed information on SL management and LEDs fixtures characteristics have been reported by Palmitessa et al. [9].

### 2.3. Plant Material and Growing Conditions

A landrace of the of Cucurbitaceae family called “Carosello leccese” (*Cucumis melo* L. subsp. melo conv. *flexuosus*) was grown for 81 days after transplant (DAT) in an extra-seasonal growth cycle with a soilless technique with close-cycle nutrient solution (NS) management. The seedlings were grown until the second true leaf stage at the plant nursery (Liuzzi Plant, Fasano-Brindisi, Italy) and transplanted in 10 L pots filled with a mixture composed of peat (Brill 3 Special, Brill Substrate GmbH & Co., Georgsdorf, Germany) and perlite (Agrilit 3, Perlite Italiana, Corsico-Milano, Italy) in a 1:1 (*v*/*v*) ratio. Ten pots were placed on each aluminum trough bench (length 3 m, width 0.26 m, slope 1%, distance between benches 1.2 m). Distance between plants on the bench was 0.30 cm with a stem density of 2.78 plants per m^−2^. The experimental layout consisted of eleven trough benches (nine benches for the experimental units and two benches as external guard rows which also included the first and last plant in each row). The principal stem of each plant was trained vertically, and the lateral stems were topped after the second node. Periodically, the oldest leaves were removed. The greenhouse temperature was controlled by natural ventilation through ridge openings set to open automatically above 20 °C. Pollination was guaranteed by the introduction of bumblebees (*Bombus terrestris* L.) into the greenhouse. Temperature and relative humidity were recorded by a CR1000 datalogger (Campbell Scientific, Logan, UT, USA). NS composition and management during the crop cycle have been extensively described by Palmitessa et al. [9]. Fruits were harvested from 25 to 81 DAT, while the biochemical analyses reported below were conducted on the fruits harvested 32 and 50 DAT. For each light treatment and replication, a sample of “Carosello leccese” fruits harvested 32 and 50 DAT was used for chemical analysis. About 120 g of fruits was freeze-dried until reaching stable weight (about 46 h) using an Alpha 2–4 LSC plus freeze-dryer (Martin Christ Gefriertrocknungsanlagen GmbH, Osterode am Harz, Germany) with a vacuum pressure of 0.015 mbar and a condenser temperature of −60 °C. The freeze-dried samples were ground at 500 µm by using a Retsch laboratory mill (Torre Boldone, BG, Italy) to obtain a homogeneous powder.

### 2.4. Chemicals

Tocopherols and polyphenols standards, as well as all HPLC grade solvents, were purchased from Sigma–Aldrich (Milano, Italy). Carotenoid standards (violaxanthin, lutein, zeaxanthin, β-cryptoxanthin, and β-carotene) were purchased from Cayman chemicals (Ann Arbor, MI, USA); antheraxanthin was tentatively identified by comparison with retention times and literature UV spectra and quantified by means of lutein standard. Chlorophylls in acetone were purchased from DHI Water & Environment (Copenhagen, Denmark)

### 2.5. Extraction and Analysis of Phenolic Compounds

Polyphenols were extracted with the methodology of Abu-Reidah et al. [29] and barely modified. Two mL of methanol 80% (*v*/*v*) was added to 100 mg of dried weight (DW) samples. They were vortexed (1 min, 3000 rpm), sonicated for 30 min in a Labsonic177 LBS1-10 ultrasonic bath (Falc Instruments, Treviglio-Bergamo, Italy), and shaken with a magnetic stirrer for 5 h. The samples were centrifuged at 4500× *g* for 10 min and the supernatants were dried through an evaporator at 40 °C. The dry residue was suspended in 100 µL of methanol 80% (*v*/*v*), filtered with 0.2 µm filters, and analyzed with HPLC as reported by Laddomada et al. [30] using a Phenomenex-luna 5 µm C18 (2) 100 Å column (250 × 4.6 mm) (Phenomenex, Torrance, CA, USA). A gradient elution program was utilized with a mobile phase consisting of acetonitrile (solution A) and 1% (*v*/*v*) H_3_PO_4_ in water (solution B) as follows: isocratic elution, 100% B, 0–30 min; linear gradient from 100% B to 85% B, 30–55 min; linear gradient from 85% B to 50% B, 55–80 min; linear gradient from 50% B to 30% B, 80–82 min; post time, 10 min before the next injection. The flow rate of the mobile phase was 1.0 mL/min, the injection volume was 20 µL and the temperature of the column was set at 30 °C. The wavelength used for the quantification of gallic acid, methyl gallate, and 3,4 dihydroxybenzoic acid was 280 nm; of *p*-coumaric acid was 295 nm; of clorogenic acid was 320 nm; and of rutin was 350 nm.

### 2.6. Extraction and Quantification of Isoprenoids

Triplicate aliquots of 500 mg DW samples were resuspended in 5 mL distilled water, thus obtaining a homogeneous suspension. Isoprenoid (tocopherols, carotenoids, and chlorophylls) extraction was performed on 500 mg of the homogeneous suspension through the method of Sadler et al. [31] as modified by Perkins-Veazie et al. [32]. Isoprenoid analyses were carried out by HPLC as described by Durante et al. [33] using an Agilent 1100 Series HPLC system equipped with a reverse-phase C30 column (5 µm, 250 × 4.6 mm) (YMC Inc., Wilmington, NC, USA). The mobile phases were: methanol (A), 0.2% ammonium acetate aqueous solution/methanol (20/80, *v*/*v*) (B), and tert-methyl butyl ether (C). The gradient elution was as follows: 0 min, 95% A and 5% B; 0–12 min, 80% A, 5% B and 15% C; 12–42 min, 30% A, 5% B and 65% C; 42–60 min, 30% A, 5% B and 65% C; 60–62 min, 95% A, and 5% B. The column was re-equilibrated for 10 min between runs. The flow rate was 1.0 mL/min, and the column temperature was maintained at 25 °C. The injection volume was 10 µL. Wavelengths used for quantification of tocopherols, carotenoids, and chlorophylls were 290 nm, 450 nm, and 665 nm, respectively.

### 2.7. Glucose and Fructose Assay and Sweetness Index

“Carosello leccese” fruit samples (the residual half part of each fruit) were freeze-dried and milled as described in Section 2.3 of this manuscript. Glucose, fructose, and sucrose content were determined by ionic chromatography (Dionex model DX500; Dionex Corp., Sunnyvale, CA, USA) using a pulsed amperometric detector (PAD) according to protocols used by Renna et al., [34]. Peak separation was performed using a Dionex CarboPac PA1 and isocratic elution with 50 mmol L^−1^ NaOH. Results were expressed as mg g^−1^ DW.

The sweetness index (SI) was calculated based on the content and sweetness properties of individual carbohydrates by multiplying the sweetness coefficient of each sugar (glucose = 1.00, fructose = 2.30 and sucrose = 1.35) by the concentration (g∙100 g^−1^ FW) of that sugar in fruits. The formula suggested by Renna et al. [22] was used:

SI = (g glucose g∙100 g^−1^ FW) × 1.00 + (g fructose g∙100 g^−1^ FW) × 2.30 + (g sucrose g∙100 g^−1^ FW) × 1.35

### 2.8. Macro and Micronutrients Analysis

Macro and microelement (Ca^2+^, K^+^, P^5+^, Mg^2+^, Na^+^, Fe^2+^, B^3+^, Mn^2+^, Ni^2+^, and Zn^2+^) concentrations were determined according to D’Imperio et al. [35]. Briefly, DW samples (obtained by placing the freshly picked fruit in a forced draft oven at 105 °C until constant weight) were mineralized with HNO_3_ at 65% (Pure grade, Carlo Erba, Cornaredo, Milano, Italy) in a microwave digestion system (MARS 6, CEM Corporation, Matthews, NC, USA). The digestion procedure was performed in two steps: 15 min to reach 200 °C and 10 min maintained at 200 °C (power set at 900–1050 W; 800 psi). The digested solutions were cooled, diluted, and filtered using a 0.45 µm filter. Samples were analyzed with an inductively coupled plasma-optical emission spectrometry (ICP-OES; 5100 VDV, Agilent Technologies, Santa Clara, CA, USA) to measure Ca^2+^, K^+^, Mg^2+^, and Na^+^ in radial mode and B^3+^, Fe^2+^, Mn^2+^, Zn^2+^, and Ni^2+^ in axial mode [36].

### 2.9. Experimental Design and Statistical Analysis

The SL treatments were arranged in a randomized block design with three replications. All data underwent analysis of variance (ANOVA) using the general linear model (GLM; SAS Software, Cary, NC, USA). The experimental factors were fixed by two-way analysis of variance (ANOVA), and the orthogonal contrasts technique was used to establish differences between means (two contrasts): (1) NL vs. LEDs; (2) (R + B) vs. (R + B + FR).

## 3. Results

### 3.1. Supplementary Light Management and Its Effects on Fruits Yield and Morphology

**Figure 2:**
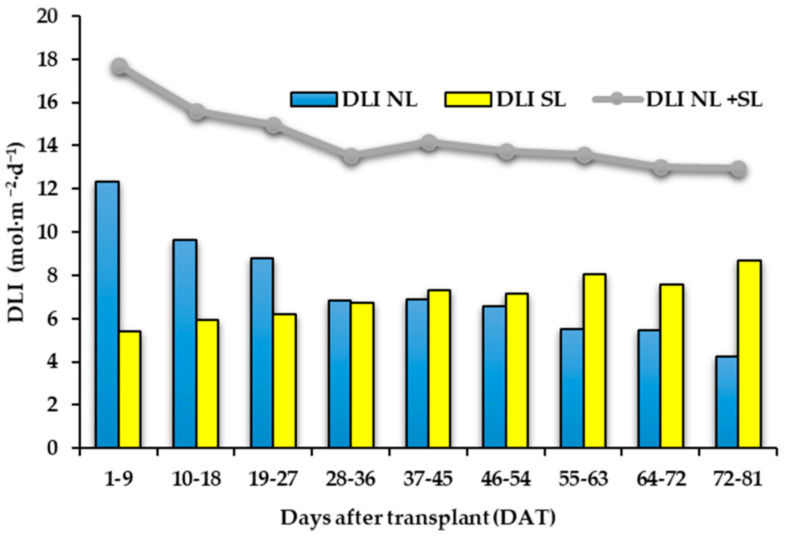
Bar charts showing the average daily light integral supplied by sun (DLI NL) and from LEDs (DLI SL) in 9-day intervals. Line graph represents the average daily light integral supplied from sun and LEDs DLI NL + SL in 9-day intervals.

Supplementary light management and light effects on “Carosello leccese” fruit yield and morphology have already been discussed by Palmitessa et al. [9]. Briefly, as reported in Figure 2, the amount of daily light integral (DLI) supplied by sun radiation to the plants was progressively reduced during the crop cycle, while the amount of DLI supplied from LEDs was increased from the start to the end of the cycle [9]. Considering cumulative production obtained 50 days after transplant (DAT), the plants grown under LEDs showed higher production than those under natural light and at the end of the crop cycle, the plants under LED showed 27% higher yield than those under natural light (3844 g∙plant^−1^ vs. 3021 g∙plant^−1^) [9]. However, the morphological characteristics of the fruits were not influenced by the treatments. The harvested fruits had an average fresh (FW) and DW of 218.7 g FW and 4.2 g·100 g^−1^ DW [9], respectively.

### 3.2. Poliphenols Profile of “Carosello leccese” Fruits

**Figure 3:**
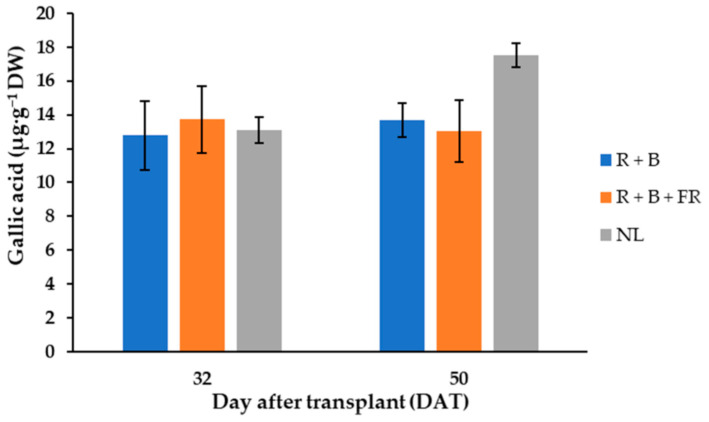
Gallic acid content of “Carosello leccese” (*Cucumis melo* L.) fruits grown under solar light (NL, no supplementary lighting) and two light spectra (see Figure 1). Values are the average of three replications. Vertical bars represent mean values ± standard error. R + B represents supplementary light with red + blue spectra; R + B + FR represents supplementary light with red + blue + far red spectra; NL represents solar light (control) treatment.

In “Carosello leccese” fruits, six phenol compounds were mainly identified, and their levels did not vary between light treatments and the days of harvest (Table 1). The polyphenol compounds were, in decreasing order: methyl gallate > gallic acid > 3,4-dihydroxybenzoic acid > *p*-coumaric > chlorogenic acid > rutin. Methyl gallate represented almost 50% of the total polyphenol content, with an average of 35.6 ± 0.7 µg·g^−1^ DW (Table 1). The second-most abundant polyphenol was gallic acid, found in the fruits harvested 32 and 50 DAT at a concentration of 13.4 ± 1.8 µg·g^−1^ DW (Table 1). Furthermore, the 50 DAT sample harvested under NL contained 23% more gallic acid than samples grown under LEDs (Figure 3). The third-most abundant polyphenol in “Carosello leccese” was 3,4-dihydroxybenzoic acid, with an average value of 7.99 ± 0.98 µg·g^−1^ DW (Table 1), followed by *p*-coumaric acid with an average content of 5.80 ± 0.59 µg·g^−1^ DW (Table 1). A content like *p*-coumaric was found for chlorogenic acid, especially for the fruits harvested 32 DAT (Table 1). For these fruits, the content of chlorogenic acid was 14.4% higher than for the fruits harvested 50 DAT. Rutin was the least abundant phenolic compound identified, with an average value of 3.78 ± 0.51 µg·g^−1^ DW (Table 1). These results indicate that, in the experimental conditions assayed, the total polyphenol content of “Carosello leccese” fruits was not affected by either DAT or SL, and the experimental conditions resulted in an average of 72.15 ± 2.0 µg·g^−1^ DW (Table 1).

### 3.3. Tocopherols and Chlorophylls Content of ‘Carosello leccese’ Fruits

The tocopherols identified in “Carosello leccese” fruits were α-tocopherol (α-T) and β-tocopherol (β-T) (Table 2). α-T was more abundant than β-T, and the levels of both compounds were influenced by DAT (Table 2). Specifically, α-T was 14% more abundant in fruits harvested 50 DAT than 32 DAT (Table 2). Moreover, the fruits obtained under NL conditions contained 26% more α-T than those under LEDs (Table 2). Instead, β-T was not influenced by light treatments, but as was observed for α-T, the fruits harvested 50 DAT had 40% more β-T compared to the fruits harvested 32 DAT (Table 2). Therefore, considering the total tocopherol content, it was found that the fruits harvested 50 DAT had 23% more compared to the fruits harvested 32 DAT (Table 2). Finally, considering the chlorophyll (Chl a + b) content, LEDs induced an increase in chlorophyll content that resulted in levels 43% higher than the level recorded in samples from plants grown under NL (Table 2). Chl a content was almost 65% of the total chlorophyll content (data not shown).

### 3.4. Carotenoids Content of “Carosello leccese” Fruits

**Figure 4:**
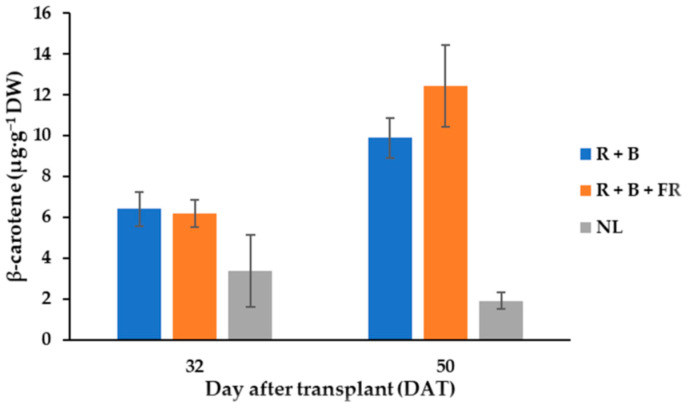
β-carotene content of “Carosello leccese” (*Cucumis melo* L.) fruits from plants grown under solar light (NL, no supplementary lighting) and two light spectra (see Figure 1). Values are an average of three replications. Bars represent mean values ± standard error. R + B represents supplementary light with red + blue spectra; R + B + FR represents supplementary light with red + blue + far red spectra; NL represents solar light (control) treatment.

Seven different carotenoid molecules were identified in fruits and their content was influenced by SL treatments (Table 3). Listed in decreasing order of concentration, the carotenoids found in fruits were: antheraxanthin > lutein > β-cryptoxanthin > β-carotene > 9 *cis*-β-carotene > violaxanthin > zeaxanthin (Table 3). Antheraxanthin content was influenced by SL treatments; indeed, after R + B + FR treatment, its value was 31% higher than the value obtained for R + B-treated plants (Table 3). R + B + FR treatment also induced a 30% higher content of lutein in respect to R + B (Table 3). The contents of β-cryptoxanthin and violaxanthin were not influenced by the supplementary light treatments and their average values were, 6.72 ± 0.90 and 3.2 ± 0.21 µg·g^−1^ DW (Table 3), respectively. Instead, especially for the fruits harvested at 50 DAT, β-carotene levels were higher under LEDs than under NL conditions (Table 3; Figure 4). In fact, the β-carotene content of the fruits grown under LEDs and harvested on 50 DAT was, on average, 11.15 µg·g^−1^ DW—that is, 61.6% higher than the value observed under NL conditions (Figure 4). Moreover, considering 50 DAT fruit samples, the R + B + FR spectrum induced a 14% higher β-carotene content than R + B treatment (Figure 4). Furthermore, 9 *cis*-β-carotene was not affected by the harvest time or light treatments and its average value was 3.37 ± 0.27 µg·g^−1^ DW. Finally, zeaxanthin was the carotenoid with the lowest concentration in fruit tissues, especially for the fruits grown under LEDs (Table 3). In the fruits obtained in NL conditions, the zeaxanthin content was 6.74 µg·g^−1^ DW—that is, higher than the value observed in the fruits harvested under LEDs. The results underwent analysis of variance as described in methods and showed that the total carotenoid content of *Carosello leccese* was not significantly influenced by light treatments or harvest time and had on average 61.7 ± 7.5 µg·g^−1^ DW (Table 3).

### 3.5. Sugars Content and Sweetness Index of “Carosello leccese” Fruits

**Figure 5:**
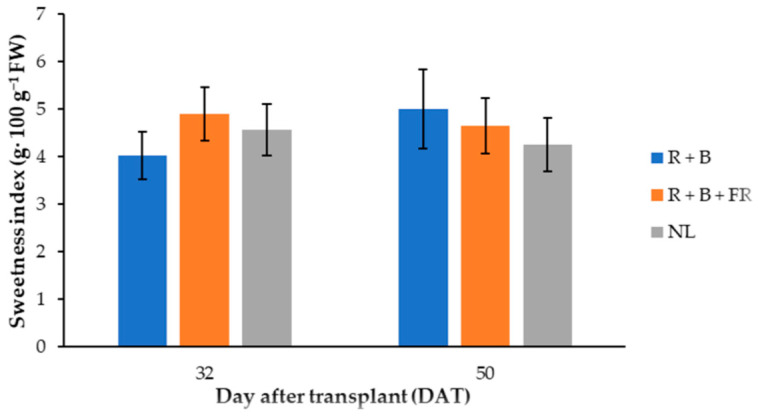
Sweetness index of “Carosello leccese” (*Cucumis melo* L.) fruits grown under solar light (NL, no supplementary lighting) and two light spectra (see Figure 1) 32 and 50 DAT. Values are an average of three replications. Vertical bars represent mean values ± standard error. R + B represents supplementary light with red + blue spectra; R + B + FR represents supplementary light with red + blue + far red spectra; NL represents solar light (control) treatment.

Fructose was the most abundant among sugars identified and it was 12% higher for the fruits harvested 32 DAT than those harvested 50 DAT (Table 4). Dissimilarly, glucose did not vary in these samples, but it was 8% higher in the fruits obtained under LEDs compared to NL conditions (Table 4). Finally, sucrose was not influenced by the treatments compared, resulting in an average of 8.0 ± 0.4 mg·g^−1^ DW (omitted data). Generally, the total sugar content in “Carosello leccese” fruits was higher when the plants were cultivated with LED modules (Table 4). In fact, the total sugar content was 7% higher for the fruits harvested under LEDs than under NL conditions (Table 4). Instead, the sweetness index (SI) had a different trend between the light treatment 32 and 50 DAT (Figure 5): 32 DAT, the SI of the peponids harvested under LEDs was similar to those of the fruits harvested under NL (Table 5). Considering light spectra, 32 DAT, the R + B + FR treatment resulted in a 17.7% higher SI than R + B. In 50-DAT-treated samples, supplemental light spectra did not induce significant SI differences (Table 5).

### 3.6. Mineral Content of Carosello leccese Fruits

The most abundant cations in “Carosello leccese” were, in descending order: potassium (K^+^) > phosphorus (P^5+^) > calcium (Ca^2+^) > magnesium (Mg^2+^) > sodium (Na^+^) > iron (Fe^2+^) > zinc (Zn^2+^) > boron (B^3+^) > manganese (Mn^2+^) > nickel (Ni^2+^) (Table 5). Potassium was always higher than 50 mg·g^−1^ DW and it was 21% higher for the fruits harvested 32 DAT than for those harvested 50 DAT (Table 5). Like potassium, phosphorus, calcium, magnesium, and iron varied between DAT and they were 26%, 27%, 33% and 13% higher, respectively, for the fruits harvested 32 DAT than 50 DAT (Table 5). Moreover, 32 DAT, the fruits obtained under control conditions had 14% less Fe^2+^ than the fruits obtained under LEDs, while 50 DAT, the fruits harvested under control conditions had 17% more Fe^2+^ than under NL treatment (Table 5). Differently, B^3+^, Mn^2+^, and Zn^2+^ did not vary between DAT and SL treatments and their average contents were 22.8 ± 1.3, 21.1 ± 1.6, and 48.2 ± 2.5 mg·kg^−1^ DW (Table 5), respectively. Finally, the least abundant cation detected was Ni^2+^, which was 40% higher for the fruits obtained under R + B than under R + B + FR (Table 5).

## 4. Discussion

### 4.1. LEDs as a Tool to Enhance Landraces of Cucumis melo L.

Recent studies have suggested that LEDs are the emerging technology to use as supplementary light in greenhouse cultivation or as a single source of light energy in indoor farming [11,37,38]. Until a few years ago, it was thought that supplementary lighting was exclusively needed during the dark season in northern region greenhouse agriculture. Currently, LED installation is also considered necessary for greenhouses in the Mediterranean Basin to maintain acceptable yields during the fall–winter period [28,39,40]. Moreover, it has been demonstrated that LEDs used as a source of supplementary light differently influenced physicochemical parameters and the antioxidant content of tomatoes [18] as well as other plant species [16].

Plants are a remarkable source of antioxidants, such as polyphenols, carotenoids, and vitamins, and they play an important role in the human diet [41]. For this reason, we are constantly looking for new products and new production techniques to obtain high-yield and high-nutritional-value plant products from out-of-season cultivations. In a previous study, we tested different cultivation techniques to enhance the production of landraces of *Cucumis melo* L. subsp. *melo* conv. *flexuous* (called “Carosello” and “Barattiere”), which are widespread in Apulia region [4,9]. The results were very satisfactory, as we observed that by using LEDs, the fruit production of “Carosello leccese” increased up to 26% compared to NL conditions [9]. In this study, we intended to investigate whether SL could positively or negatively affect the profile of bioactive compounds of “Carosello leccese” fruits.

### 4.2. Polyphenol Fruit Profiles

Polyphenols are known for their ability to scavenge free radicals through hydrogen atom transfer, single electron transfer, and/or chelation of metal cations to contribute to the plant defense system against biotic and abiotic stresses and are also important for human health [42]. Our experiments confirmed that the landraces of *Cucumis melo* L. have a rich polyphenols profile [43], and in the case of “Carosello leccese”, methyl gallate, gallic acid, 3,4-dihydroxybenzoic acid, *p*-coumaric, chlorogenic acid, and rutin are the main phenolic compounds identified (Table 1). Our results showed that the polyphenols profile of “Carosello leccese” was not influenced by light treatments (Table 1). However, it cannot be excluded that further increasing of light intensity or photoperiod length could lower polyphenols as was reported for green barley (*Hordeum vulgare* L.) by Kowalczewski et al. [44]. Methyl gallate and gallic acid are gallotannins and methyl gallate is a methyl ester of gallic acid. These molecules were found in higher concentrations in plants such as including pomegranate and mango fruits and are known to have important biological activities [45,46]. Gallic acid inhibits the growth of several pathogenic bacteria [47] and is reported to have antioxidant and anti-inflammatory properties, protecting human cells against oxidative stresses [48]. Both gallic acid and methyl gallate have also been demonstrated to possess antioxidative and antiviral effects in vitro and in vivo [49,50,51]. Methyl gallate is also a powerful and highly specific inhibitor of herpes simplex virus [49]. 3,4-dihydroxybenzoic acid, *p*-coumaric acid, and chlorogenic acid have antioxidant and antiradical activity [52]. Rutin has antiplatelet, antiviral, and antihypertensive properties, and also strengthens capillaries due to its high radical scavenging and antioxidant capacity [53,54]. Due to these important health effects of the above reported compounds, the consumption of “Carosello leccese” fruits should be further encouraged. To this aim, it is thus important to ascertain if the use of supplementary LED light in out-of-season cultivation cycles could have effects on the polyphenolic profile of *Carosello leccese* fruits. The results obtained showed that polyphenol profile was not influenced by supplementary light of the LEDs, thus encouraging the use of this technology in out-of-season cultivation cycles.

### 4.3. Tochopherols and Clorophylls Fruits Profile

Tocopherols usually occur in one of four forms, namely α-, β-, γ-, and δ-tocopherol, differing only in the number and position of methylation on the chromanol ring. The number and position of methyl groups of tocopherols influence their ease of donating hydrogen, hence their antioxidant effectiveness. They are well recognized for their effective inhibition of lipid oxidation in foods and biological systems [55]. Since vitamin E is only synthesized by plants, it is a very important dietary nutrient for humans and animals [56]. A-T is the most biologically active form of vitamin E [18] and it is generally agreed that the relative antioxidant activity of the tocopherols in vivo is in the order α > β > γ > δ [56]. In “Carosello leccese” fruits, α-T was 70–80% of the total tocopherols (Table 3), confirming that this landrace is an important source of substances with bioactive action on the human organism. Differently from what was observed for tomatoes [18], LED supplementary light reduced the α-T content of “Carosello leccese”, while the total content of tocopherols was not influenced by light treatments (Table 3). Considering the samples harvested 32 and 50 DAT, the highest tocopherol content was found in the fruits harvested 32 DAT (Table 3). Considering this, our hypothesis is that the increasing time of exposure to LEDs could result in a decrease in tocopherols (Table 3). In agreement with these results, in a previous work, it has been reported that the blue light supplied by LEDs resulted in a reduction of tocopherol content in mustard, beet, and parsley microgreens [57]. Inversely to tocopherols, chlorophyll content increased under LED supplementary light treatments (Table 3). Like in cucumber, the fruit skin’s chlorophyll content is an important quality factor of “Carosello leccese” fruits, which strongly influences the keeping quality of the fruit [58]. In a previous study conducted on cucumber, it was shown that LEDs induced an increase in the chlorophyll content of the fruit skin, causing an increase in the total chlorophyll content [58], as was also observed for “Carosello leccese” in this study (Table 3).

### 4.4. Carotenoids Fruits Profile

Carotenoids are known to be very efficient physical and chemical quenchers of singlet oxygen, as well as potent scavengers of other reactive oxygen species, enhancing the immune response, suppressing cancer development, and protecting eye tissues [59]. β-carotene and β-cryptoxanthin are pro-vitamin-A carotenoids with a protective action against cardiovascular diseases, while violaxanthin, antheraxanthin, zeaxanthin, and lutein protect the macula from light-induced damage [17]. In melons, fruit development and ripening is associated with a decrease in chlorophyll and an increase in carotenoid content, but, being harvested at immature stage, “Carosello leccese” has a higher chlorophyll than carotenoid content [9]. In this study no significant correlation was observed between supplementary light and carotenoid content, though it was found that R + B + FR treatment induced a higher content of total carotenoids (Table 3). Previous studies have reported the effects of FR radiation on carotenoid content in tomato fruits [60]. This confirms the results obtained in our experiment, where lutein and antheraxanthin were more abundant under R + B + FR radiation than under other light treatments (Table 3). Very interesting was the influence of supplementary light on zeaxanthin content: the application of LEDs as a source of supplementary light almost cleared the content of zeaxanthin in “Carosello leccese” fruits (Table 3). This trend could probably be linked to a photoinhibition process caused by LED light intensity. Furthermore, LED treatment caused lower concentrations of zeaxanthin + anteraxanthin + violaxanthin (xanthophyll cycle pigments). Thus, it is likely that the LED treatments did not contribute additional stress to photosystem II (PSII) light harvesting complexes that have a role in photosynthetic energy collection. Indeed, to avoid photo-oxidative damage of the photosynthetic apparatus due to the formation of reactive oxygen species under excess light, xanthophylls are involved either directly or indirectly in the non-photochemical quenching of excess light energy at the acceptor side of PSI or at PSII [61].

### 4.5. Sweetness Index, Sugars and Mineral Elements Fruits Profile

**Figure 6:**
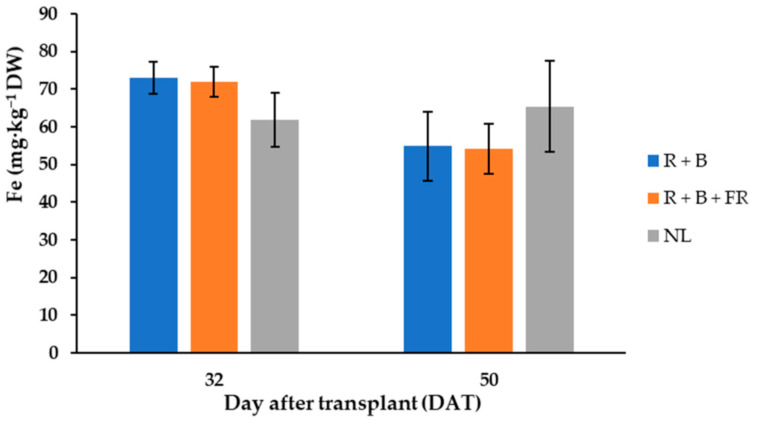
Iron (Fe) content, expressed as mg·kg^−1^ D.W. of “Carosello leccese” (*Cucumis melo* L.) fruits grown under solar light (NL, no supplementary lighting) and two light spectra (see Figure 1). Values are an average of three replications. Vertical bars represent mean values ± standard error. R + B represents supplementary light with red + blue spectra; R + B + FR represents supplementary light with red + blue + far red spectra; NL represents solar light (control) treatment.

Sweetness Index (SI) was calculated to determine the sweetness perception of fruits [62]. In this research, it was observed that the SI varied between light conditions and the different DAT of the samples (Table 4; Figure 6). However, considering the SI values found for “Barattiere” (another Apulian landrace of *C. melo* L.) [22], “Carosello leccese” fruits had lower SI independent of light treatment and harvest time (Table 4). Since “Carosello leccese” looks like a melon, but is consumed in the same way as cucumber, it might be interesting to the compare sugar content of this local variety with those of cucumber and melon. Based on the average data reported by the National Nutrient Database of the United States Department of Agriculture, cucumber contains 6.3 g∙kg^−1^ of FW of glucose and 7.5 g∙kg^−1^ FW of fructose [63], while melon (cantaloupe type) contains 15.4 g∙kg^−1^ FW of glucose, 18.7 g∙kg^−1^ FW of fructose [64]. Considering a DW content of 42 g∙kg^−1^ of FW [9], the glucose content of “Carosello leccese” grown under LED was 9.43 g∙kg^−1^ FW, while under NL it was 8.61 g∙kg^−1^ FW (Table 4). Therefore, the glucose content of “Carosello leccese” was higher than cucumber, but lower than cantaloupe and “Barattiere” [22]. Instead, fructose content in “Carosello leccese” was different between 32 and 50 DAT samples; it was 13.1 and 11.5 g∙kg^−1^ FW (Table 4), respsectively. This was higher than in cucumber but lower than in cantaloupe and “Barattiere” [22]. Finally, sucrose content in “Carosello leccese” fruits was very low (around 0.336 g∙kg^−1^ FW) and was not influenced by supplementary light treatment. This was another confirmation that the fruits of “Carosello leccese” were harvested at an immature stage. [63].

Considering the mineral element content, the highest values were found in the fruits harvested 32 DAT and generally light treatments did not affect their concentrations (Table 5) despite different studies reporting that light treatments can modify mineral profiles [18,65]. Like cucumber, cantaloupe, and “Barattiere”, “Carosello leccese” is an important source of mineral elements for human organisms. Potassium was the mineral element most abundant in “Carosello leccese” fruits and its content on 32 DAT was 298 mg∙kg^−1^ FW (Table 5). This was higher than K^+^ content in cantaloupe (267 mg∙kg^−1^ FW; [64]), in cucumber (136 mg∙kg^−1^ FW; [63]), and in “Barattiere” (217 mg∙kg^−1^ FW; [22]). The second-most abundant element in “Carosello leccese” was phosphorus; in the fruits harvested 32 DAT, it was 54.8 mg∙kg^−1^ FW (Table 5). This was higher than P^5+^ content in cantaloupe (15 mg∙kg^−1^ FW; [64]) and in cucumber (21 mg∙kg^−1^ FW; [63]). The third-most abundant mineral element in “Carosello leccese” was calcium (Ca^2+^) and its content 32 DAT was 35.5 mg∙kg^−1^ FW (Table 5). This was higher than Ca^2+^ content in cantaloupe (9 mg∙kg^−1^ FW; [64]), in cucumber (14 mg∙kg^−1^ FW; [63]), and in “Barattiere” (22.4 mg∙kg^−1^ FW; [22]). The fourth-most abundant mineral element found in “Carosello leccese” was magnesium (Mg^2+^); 32 DAT, it was 17.7 mg∙kg^−1^ FW (Table 5). This was higher than Mg^2+^ content in cantaloupe (12 mg∙kg^−1^ FW; [64]), in cucumber (12 mg∙kg^−1^ FW; [63]), and in “Barattiere” (14.8 mg∙kg^−1^ FW; [22]). Finally, sodium (Na^+^) was another important element contained in “Carosello leccese” fruit tissues, and it was on average 4.96 mg∙kg^−1^ FW (Table 5). This was lower than in cantaloupe (16.0 mg∙kg^−1^ FW; [64]), but it was higher compared to cucumber (2.0 mg∙kg^−1^ FW; [63]) and “Barattiere” (2.7 mg∙kg^−1^ FW; [22]) cultivated in open air. From a nutritional standpoint, it is important to highlight that a high intake of Na^+^ may increase the risk of some diseases; in fact, the nutritional recommendation is to not exceed 2 g/day. The results of this study show that 100 g of this product only supplied 0.49 mg of Na, which is negligible with respect to the recommended limits. Therefore, “Carosello leccese” is an important source of substances with bioactive functions and a very important source of mineral elements (more so than cucumber). In addition, given that the global population is expected to grow from 7 to 9 billion by 2050, there will be rapidly increasing demand for vegetable products. Doing more with less is imperative. Our results indicate that light technology could be used as an innovative method for increasing the yield [9] without negative alteration of the mineral quality of fruits.

## 5. Conclusions

“Carosello leccese”, an Apulian landrace of *Cucumis melo* L. subsp. *melo* conv. *Flexuosus*, is well suited to greenhouse cultivation. To increase the cultivation of “Carosello leccese”, LEDs have been proposed as a system to obtain high yield through out-of-season production [9]. The results obtained with this study show that LEDs allow an increase in yield without negatively affecting the bioactive content in the fruits. Based on our knowledge, this is the first study that reports the nutritional profile of the bioactive substances contained in “Carosello leccese”. The peponids of this species are rich in molecules with bioactive actions for human organisms (methyl gallate, α-tocopherol, fructose, and antheraxanthin). Moreover, it has been shown that LEDs can increase the content of α-tocopherol, chlorophylls, β-carotenes, glucose, and total sugar. However, further studies will be conducted on “Carosello leccese” fruits to better understand the influence of LEDs on zeaxanthin content, which was almost reset applying the supplementary light technique.

## Figures and Tables

**Table 1:** Effects of the day of harvest and light treatments on the polyphenols profile of the fruits of a landrace of *Cucumis melo* L. (“Carosello leccese”). Values are the average ± DS of three replications.

	Gallic Acid	3,4-dihydroxybenzoic Acid	Methyl Gallate	*p*-Coumaric Acid	Chlorogenic Acid	Rutin	Total
	µg·g^−1^ DW						
Day after transplant (DAT)							
32	12.1 ± 2.6	7.29 ± 0.99	35.0 ± 5.2	5.96 ± 0.78	5.81 ± 0.66	4.15 ± 0.50	70.7 ± 8.2
50	14.7 ± 2.8	8.69 ± 1.18	36.1 ± 5.5	5.63 ± 0.53	4.97 ± 0.67	3.41 ± 0.75	73.6 ± 6.7
Light treatment							
Control (NL)	15.3 ± 2.5	8.00 ± 0.96	37.4 ± 3.4	5.59 ± 0.82	5.44 ± 1.16	3.47 ± 0.87	75.2 ± 6.0
R + B + FR	11.8 ± 2.9	8.13 ± 2.01	37.0 ± 6.7	5.49 ± 0.32	5.08 ± 0.52	3.62 ± 0.63	71.7 ± 9.9
R + B	13.2 ± 2.7	7.83 ± 0.73	32.4 ± 4.2	6.30 ± 0.55	5.65 ± 0.50	4.23 ±0.48	69.6 ± 5.8
Significance ^1^							
DAT	ns	ns	ns	ns	**	ns	ns
NL vs. LEDs	ns	ns	ns	ns	ns	ns	ns
R + B vs. R + B + FR	ns	ns	ns	ns	ns	ns	ns
Date * (NL vs. LEDs) ^2^	*	ns	ns	ns	ns	ns	ns
Date * (R + B vs. R + B + FR)^2^	ns	ns	ns	ns	ns	ns	ns

^1^ Significance: ns = not significant; * and ** significant for *p* ≤ 0.05 and *p* ≤ 0.01 respectively. ^2^ The significant interactions were reported in figures.

**Table 2:** Tocopherol and chlorophyll content of the fruits of a landrace of *Cucumis melo* L. (“Carosello leccese”) sampled 32 or 50 days after transplant (DAT) from plants subjected to different light treatments. Values are average ± SD of three replications.

	Tocopherols			Chlorophylls
	α-T	β-T	Total	Chl a + b
	µg·g^−1^ DW			µg·g^−1^ DW
Day after transplant (DAT)				
32	7.7 ± 1.4	2.66 ± 1.05	10.4 ± 2.1	54 ± 12
50	9.0 ± 2.1	4.40 ± 1.37	13.4 ± 2.0	74 ± 22
Light treatment				
Control (NL)	10.1 ± 1.4	3.16 ± 1.36	13.3 ± 2.4	42 ± 6
R + B + FR	7.4 ± 1.4	3.97 ± 1.11	11.4 ± 1.9	78 ± 12
R + B	7.5 ± 1.4	3.46 ± 2.01	10.9 ± 3.0	72 ± 22
Significance ^1^				
DAT	*	**	*	ns
NL vs. LEDs	*	ns	ns	**
R + B vs. R + B + FR	ns	ns	ns	ns
Date * (NL vs. LEDs)	ns	ns	ns	ns
Date * (R + B vs. R + B + FR)	ns	ns	ns	ns

^1^ Significance: ns = not significant; * and ** significant for *p* ≤ 0.05 and *p* ≤ 0.01 respectively.

**Table 3:** Carotenoid profiles of the fruits of a landrace of *Cucumis melo* L. (“Carosello leccese”) from plants grown under solar light (NL, no supplementary lighting) and two different light spectra (see Figure 1). Values are average ± SD of three replications.

	Violaxanthin	Antheraxanthin	Lutein	Zeaxanthin	β-cryptoxanthin	β-carotene	9 *cis*-β-carotene	Total
	µg·g^−1^ DW							
Day after transplant (DAT)								
32	2.84 ± 0.33	19.8 ± 4.5	13.3 ± 4.0	2.00 ± 3.01	6.50 ± 1.02	5.31 ± 0.89	3.37 ± 0.39	53 ± 10
50	3.49 ± 0.38	27.8 ± 8.8	17.7 ± 4.5	2.71 ± 3.86	6.93 ± 0.86	8.07 ± 1.12	3.50 ± 0.44	70 ± 14
Light treatment								
Control (NL)	3.35 ± 0.22	25.3 ± 6.3	15.4 ± 3.0	6.74 ± 2.05	7.47 ± 0.45	3.23 ± 0.74	3.67 ± 0.53	65 ± 5
R + B + FR	3.21 ± 0.65	27.3 ± 3.1	18.3 ± 6.5	0.20 ± 0.05	5.94 ± 0.76	9.29 ± 1.89	3.13 ± 0.61	67 ± 11
R + B	2.93 ± 0.46	18.8 ± 10.9	12.9 ± 2.3	0.13 ± 0.06	6.73 ± 0.91	8.15 ± 1.44	3.32 ± 0.65	53 ± 6
Significance ^1^								
DAT	ns	ns	ns	ns	ns	ns	ns	ns
NL vs. LEDs	ns	ns	ns	***	ns	*	ns	ns
R + B vs. R + B + FR	ns	*	*	ns	ns	ns	ns	ns
Date * (NL vs. LEDs) ^2^	ns	ns	ns	ns	ns	*	ns	ns
Date * (R + B vs. R + B + FR) ^2^	ns	ns	ns	ns	ns	*	ns	ns

^1^ Significance: ns = not significant; * and *** respectively significant for *p* ≤ 0.05 and *p* ≤ 0.001. ^2^ The significant interactions were reported in figures.

**Table 4:** Sugar content of the fruits of a landrace of *Cucumis melo* L. (“Carosello leccese”) in fruits from plants grown under solar light and two different light spectra. Values are average ± SD of three replications.

	Glucose	Fructose	Total Sugars ^3^	Sweetness Index
		mg·g^−1^ DW		g·100 g^−1^ FW
Day after transplant (DAT)				
32	215 ± 19	313 ± 29	528 ± 42	4.61 ± 0.22
50	221 ± 22	274 ±27	495 ± 43	4.76 ± 0.34
Light treatment				
Control (NL)	205 ± 13	281 ± 18	487 ± 31	4.53 ± 0.24
R + B + FR	219 ± 16	310 ± 18	529 ± 29	4.89 ± 0.34
R + B	229 ± 14	290 ± 53	519 ± 62	4.63 ± 0.20
Significance ^1^				
DAT	ns	*	ns	ns
NL vs. LEDs	*	ns	*	ns
R + B vs. R + B + FR	ns	ns	ns	ns
Date * (NL vs. LEDs) ^2^	ns	ns	ns	*
Date * (R + B vs. R + B + FR) ^2^	ns	ns	ns	**

^1^ Significance: ns = not significant; * and ** significant for *p* ≤ 0.05 and *p* ≤ 0.01 respectively. ^2^ The significant interactions were reported in figures where the outcome of the ANOVA was not shown. ^3^ Sucrose was on average equal to 7.99 mg·g^−1^ DW.

**Table 5:** Effects of the day of harvest and light treatments on the mineral content of the fruits of a landrace of *Cucumis melo* L. (“Carosello leccese”). Values are average ± SD of three replications.

	Ca^2+^	K^+^	P^5+^	Mg^2+^	Na^+^	Fe^2+^	B^3+^	Mn^2+^	Ni^2+^	Zn^2+^
	mg·g^−1^ DW					mg·kg^−1^ DW				
Day after transplant (DAT)										
32	8.47 ± 1.02	71.0 ± 6.0	13.06 ± 1.96	4.21 ± 0.54	1.18 ± 0.23	67.7 ± 7.1	22.7 ± 2.6	22.0 ± 3.2	1.09 ± 0.38	52.4 ± 7.7
50	6.21 ± 0.85	55.9 ± 5.9	9.61 ± 1.00	2.83 ± 0.39	1.28 ± 0.32	58.1 ± 9.8	22.8 ± 4.4	20.2 ± 3.1	1.11 ± 0.44	44.0 ± 7.9
Light treatment										
Control (NL)	6.89 ± 0.69	65.8 ± 7.8	12.36 ± 3.02	3.45 ± 1.12	1.08 ± 0.08	63.7 ± 9.1	21.8 ± 3.6	22.2 ± 3.9	1.17 ± 0.31	49.8 ± 5.1
R + B + FR	7.24 ± 0.45	60.9 ± 6.7	11.50 ± 1.78	3.55 ± 0.53	1.54 ± 0.22	61.1 ± 9.6	24.3 ± 3.6	21.7 ± 3.8	0.80 ± 0.30	45.3 ± 7.6
R + B	7.90 ± 1.18	63.8 ± 6.8	10.14 ± 1.78	3.51 ± 0.92	1.08 ± 0.16	63.9 ± 9.8	22.2 ± 3.4	19.3 ± 3.4	1.33 ± 0.44	49.5 ± 7.4
Significance ^1^										
DAT	*	*	*	**	ns	*	ns	ns	ns	ns
NL vs. LEDs	ns	ns	ns	ns	ns	ns	ns	ns	ns	ns
R + B vs. R + B + FR	ns	ns	ns	ns	*	ns	ns	ns	*	ns
Date * (NL vs. LEDs) ^2^	ns	ns	ns	ns	ns	*	ns	ns	ns	ns
Date * (R + B vs. R + B + FR) ^2^	ns	ns	ns	ns	ns	ns	ns	ns	ns	ns

^1^ Significance: ns = not significant; * and ** significant for *p* ≤ 0.05 and *p* ≤ 0.01 respectively. ^2^ The significant interactions were reported in figures.

## Data Availability

The raw data supporting the conclusions of this article will be made available by the authors, without undue reservation.
